# Three-dimensional soft tissue changes after simulated protrusion of upper and lower incisors in young adults: an experimental study

**DOI:** 10.1186/s13005-025-00561-2

**Published:** 2025-11-27

**Authors:** Julia C. Glöggler, To Mai Pham, Falko Schmidt, Stefan Repky, Bernd G. Lapatki

**Affiliations:** 1 Department of Orthodontics, Ulm University, Albert-Einstein-Allee 11, Ulm, 89081 Germany; 2 Institute of Statistics, Ulm University, Helmholtzstraße 20, Ulm, 89081 Germany

**Keywords:** Perioral soft tissue change, Simulated advancement, Lips, Alignment, 3D, Incisor position, Landmarks

## Abstract

**Background:**

One component of facial aesthetics is a harmonic facial profile. Orthodontic treatment and/or orthognathic surgery can affect the facial profile by influencing the underlying hard-tissue structure. The aim of this study was to improve the prediction of treatment outcomes by evaluating 3D perioral frontal and buccal soft-tissue changes after protrusion of incisors in ten young adults.

**Methods:**

The participants had neutral occlusion and physiologic incisor inclination. 3D-printed acrylic veneers with thicknesses of 1–4 mm were bonded to the labial surfaces of the upper and lower incisors. 3D face scans with and without veneers were taken to evaluate perioral soft tissue changes using 44 landmarks. Face scans were superimposed at the forehead. Soft-tissue changes were evaluated using linear mixed-effects models.

**Results:**

Interrelations between simulated incisor protrusion and the resulting soft-tissue changes were statistically highly significant (*p* < 0.005) and nearly linear. Simulated isolated upper incisor protrusion caused greatest forward positioning of the labrale superius convex [LS-con] landmark—by 0.65 mm per millimetre of veneer thickness. Lower lip displacement at the labrale inferius convex [LI-con] landmark was greatest when both upper and lower incisors were protruded (0.76 mm/mm). The vertical lip displacement rate for simulated upper and lower incisor protrusion was 0.12 mm/mm upward positioning of LS-con and 0.66 mm/mm downward positioning of LI-con. Mixed-effects models revealed that a 1-mm increase in initial lip thickness at the labrale superius reduces soft-tissue displacement by about 8% of the simulated tooth movement.

**Conclusions:**

Anterior positioning of incisors leads to predictable, direction-specific changes in perioral soft tissues. The magnitude and pattern of soft-tissue responses vary with the combination of upper and lower incisor movement and individual lip thickness. These findings may support clinical planning in orthodontic and/or orthognathic treatments.

## Background

Facial aesthetics are determined by the harmonic relationship between underlying hard and soft tissues [1, 2]. This relationship can be affected by orthodontic treatment and/or orthognathic surgery, which alter the contours of perioral soft tissue by changing the underlying hard tissue of the mandibular and maxillary jaw bases and the dentoalveolar complex [2]. The lip profile and lip prominence are particularly affected by incisor movements [3].

Increasing attention has been paid to how orthodontic treatment influences facial soft tissue [4]. To achieve this, orthodontic treatment needs to be planned with a deeper comprehension of how changes to hard tissues affect soft tissues [4, 5]. However, how hard-tissue movements affect perioral soft tissues is difficult to predict and varies considerably between individuals [6–9]. This is because of the complex interplay between hard tissues and soft-tissue variables such as lip thickness, the presence and thickness of subcutaneous fat, the architecture of perioral muscle subcomponents, and the mechanical characteristics of skin and connective tissue [3, 10–14].

Changes to facial soft tissues and facial aesthetics during orthodontic and/or orthognathic treatment can be evaluated by 3D facial imaging using optical techniques [4]. These 3D imaging techniques have multiple advantages over 2D techniques, which provide limited information and show 3D geometry on a 2D plane [15]. These advantages include being able to take relatively precise measurements faster and non-invasively [4], and different study models have used these techniques to determine the quantitative interrelations between changes in perioral hard and soft tissues; for example, some studies have used 3D stereophotogrammetry [16, 17] or laser scanning [18] to investigate soft-tissue changes after bracket debonding.

Beyond diagnostics, such imaging technologies are now integral to digital treatment workflows. In modern dentistry, digital tools such as cone-beam computed tomography (CBCT), intraoral and facial scanning, and treatment planning software enable precise, interdisciplinary approaches—such as the simultaneous orthodontic and prosthetic planning of specific tooth positions. This digital workflow has opened new possibilities for orthodontists to fine-tune tooth positioning with both functional and aesthetic goals in mind. Our study aims to support these digital workflows by quantifying 3D perioral soft-tissue changes following simulated incisor protrusion. To achieve this aim, we used high-resolution 3D facial scans and a dense landmark protocol to precisely analyse soft-tissue changes.

Earlier studies that employed 3D imaging to investigate soft-tissue changes—such as those analysing bracket debonding—found that the oral commissures and lower lips retruded after debonding [16–18] whereas the morphology and position of the upper lips did not change [16]. However, there were limitations to these studies; only a small part of the labial crown surface was covered, and the incisor position was changed in one small step (corresponding to the vestibulo-oral bracket thickness) rather than stepwise. Other studies using 3D imaging have quantified the effects of retracting the upper and lower incisor segments after premolar extraction in patients with anterior crowding [14, 19–23]. For instance, cephalometric analysis showed that retraction of both upper and lower incisors by 1 mm retracts both the upper and lower lips by 44% [20]. Other studies have reported even greater changes in the horizontal position of the upper lips (41–53%) and the lower lips (22–82%) after hard-tissue changes [8, 19]. Significant vertical movements of the lips have also been observed [19], including inferior movement of the upper lip and superior movement of the lower lip due to incisor retrusion after first premolar extractions [19].

These clinical study models are limited by the relative positional changes of incisor segments relative to the perioral soft tissues occurring over a long period. This means the observed effects may be biased by natural temporal soft-tissue changes such as those induced by growth, aging, or weight changes. Moreover, measuring changes over time requires the monitoring of distinct landmarks, but it is not ethical or practical to mark landmarks on the skin for several months of study. Instead, only distinct anatomical features or reconstructed landmarks can be monitored. This makes it very difficult to measure changes in the perioral area precisely. Another limitation of these previous studies is that most of the data were based on lateral cephalometric radiographs, which do not provide information beyond the midsagittal projection as provided by 3D imaging devices [20, 22].

These limitations can be overcome with an alternative study model that experimentally simulates vestibulo-oral changes in incisor positions using the labial crown surfaces to support the positioning of additional artificial hard tissue. Recent studies have simulated protrusion of the upper incisors [6, 24] and the upper anterior teeth from canine to canine [9] using wax [6], an acrylic resin veneer [9], and films [24] of different thicknesses. As expected, most perioral landmarks identified using 3D stereophotogrammetry showed forward and upward movements [6, 24] that were greatest in the sagittal direction, followed by the vertical and transverse directions [6]. Simulating protrusion of the upper anterior teeth caused significant forward protrusion of the upper and lower vermilion [9].

To date, perioral soft-tissue changes have been evaluated in experimental models using simulated protrusion of the upper incisors only [6, 9, 24]. This means that the corresponding interrelation between the lower incisor segment and lower lip as well as a possible mutual influence of both upper and lower incisor protrusion has not been quantified. To address this gap, we evaluated how the perioral soft tissue and buccal region responded to simulated isolated and combined stepwise protrusion of maxillary and/or mandibular incisors. We hypothesized that changes in incisor position would affect lip profile and prominence differently in the upper and lower jaw, based on anatomical and possibly biomechanical differences between the upper and lower lip tissues. We also hypothesized that these interrelations depend on individual lip thickness, which may explain the large variability observed in previous studies.

## Materials and methods

Ten healthy participants (5 women and 5 men) with an average age of 25 ± 1.7 years (range: 21–27 years), all of Caucasian origin, were enrolled in this experimental study. Inclusion criteria included neutral occlusion of the first molars, defined as a maximum deviation from class I relationships of a quarter-step mesio- or distal occlusion. Further inclusion criteria were an overjet of 1–3 mm, a vertical overbite of 1–3 mm, and presence of all teeth (except third molars) with either no crowding or mild crowding. Exclusion criteria were ongoing orthodontic treatment, prosthetic tooth reconstructions, cleft lip or palate, craniofacial syndromes, and lip incompetence or asymmetries in the lower face (such as lip-line canting or a laterognathic mandible ≥ 3 mm).

The study protocol was approved by the Ethics Committee of Ulm University (approval no. 249 − 12), and informed written consent was obtained from all participants before the investigation.

**Fig. 1:**
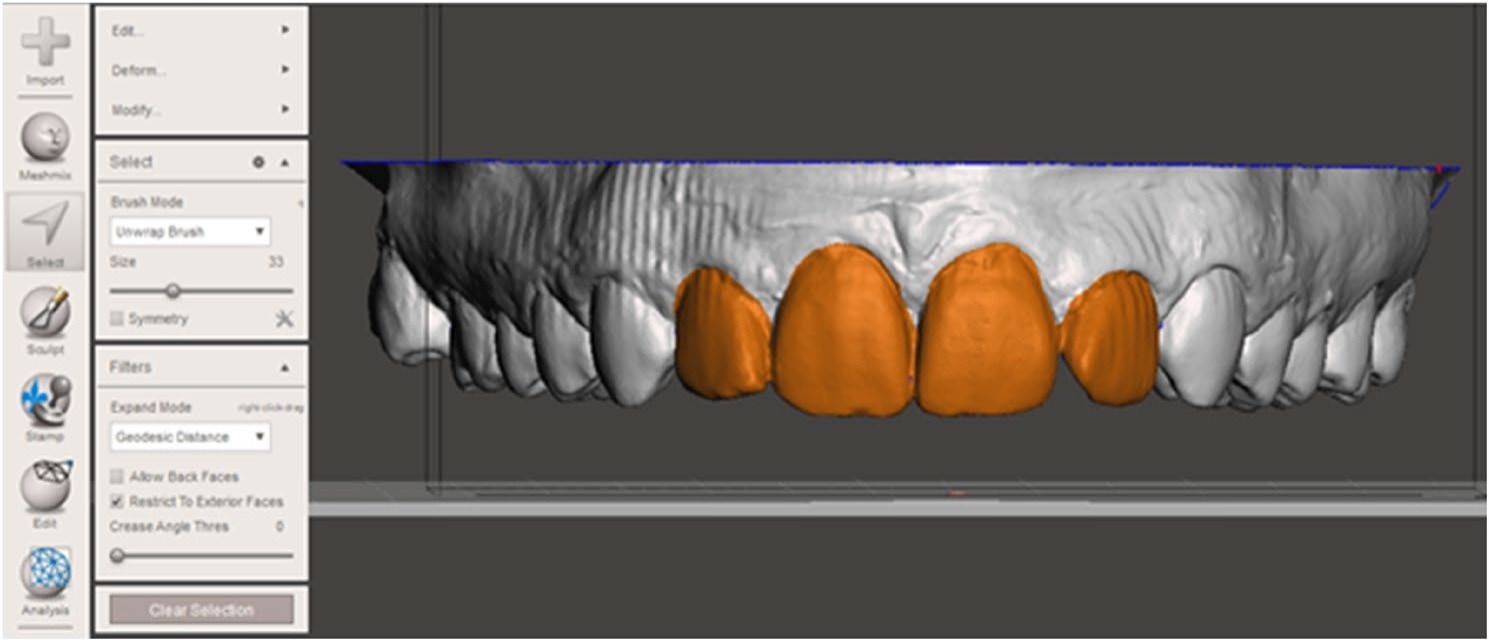
Computer-aided design of the acrylic veneers covering the entire labial crown surface of the four maxillary incisors using Meshmixer software (Autodesk Inc, Mill Valley, USA)

Before the experiments were performed, dental impressions were taken with plaster casts. These casts were digitized using a structured-light scanner (d-STATION3D, Breuckmann, Meersburg, Germany). Corresponding dental arch models were imported into Meshmixer software (Autodesk Inc, Mill Valley, USA) to design veneers that covered the entire labial crown surface of the four maxillary and mandibular incisors (Fig. 1). 

**Fig. 2:**
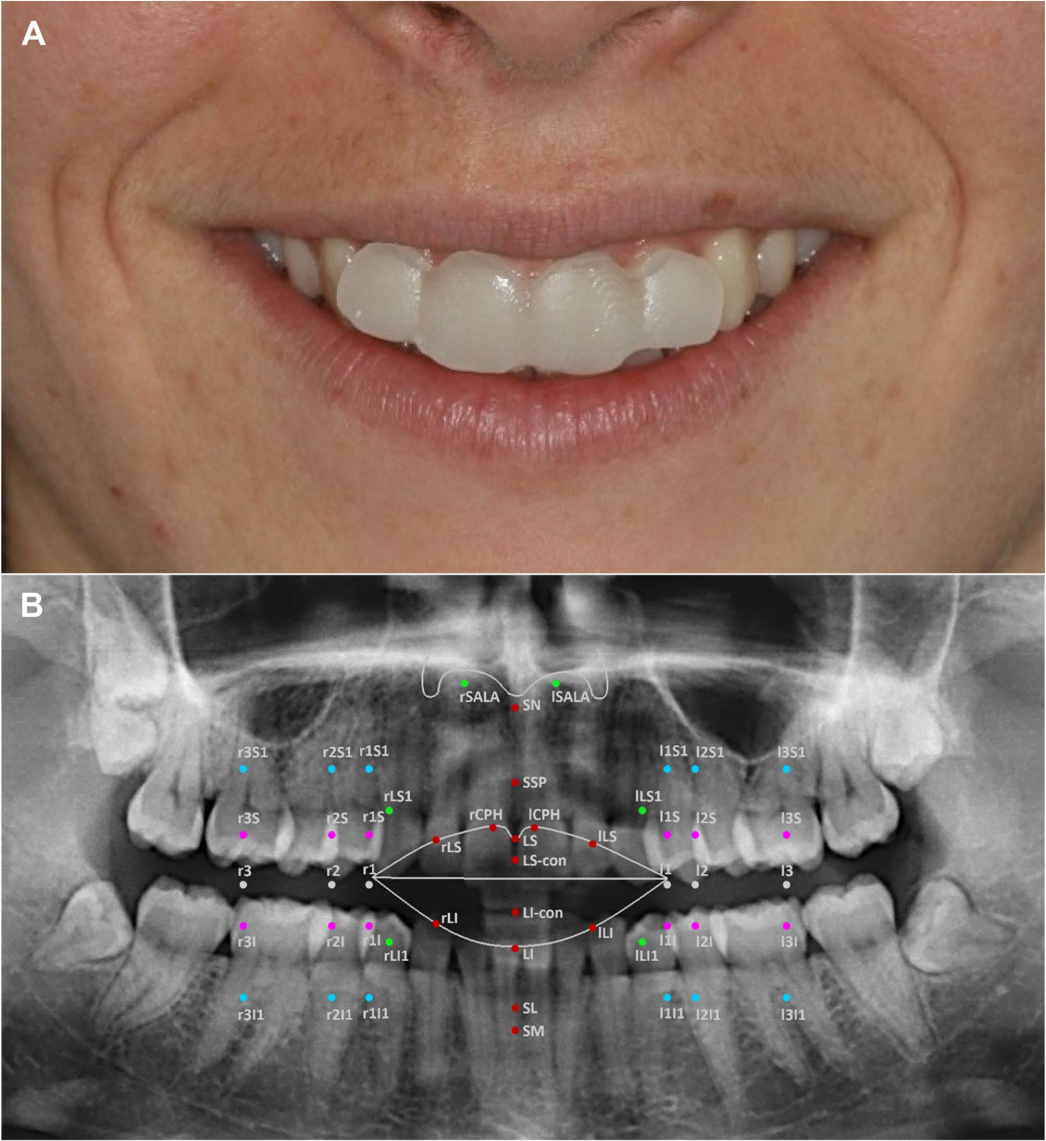
**A** Temporary placement of 3D printed acrylic veneers on the labial surface of the maxillary incisors to simulate upper incisor protrusion; the extent of 4-mm veneer thickness is visible at the gingival margin. **B** Orthopantomogram showing a schematic overview of the position of the 44 landmarks in relation to the underlying hard tissue structures. Each landmark is described in detail in Table 1

Maxillary veneers were designed to end exactly at the level of the incisal edges. Lower veneers were vertically reduced in the incisal region to avoid any precontacts in static occlusion (maximal intercuspal position) and during dynamic occlusion (mandibular movements such as protrusion or lateral excursions). Veneers with uniform thicknesses of either 1-, 2-, 3-, or 4 mm were designed for the upper and lower frontal segments in each participant. Corresponding digital models were exported and fabricated using a resin-based 3D printer (Imprimo^®^ 90, Imprimo LC Splint printing material, Scheu Dental, Iserlohn, Deutschland) (Fig. 2A).

3D facial scans including the left and right Tragi were taken using a structured-light colour scanner (optoTop-HE, Breuckmann, Meersburg, Germany) with a resolution of 1284 × 1036 pixels, an accuracy of ± 0.2 mm, and an acquisition speed of 0.8 s per scan. All experiments were performed by a single investigator (T.M.P.).

First, patients were seated comfortably in an upright position and asked to hold their head in a natural position with a neutral facial expression. The 44 landmarks (Fig. 2B and Table 1) were marked in the perioral region using a hypoallergenic cosmetic pencil [25].

**Table 1:** Location and definition of landmarks

**Landmarks (N = 44)**			**Definition**
Along the lip contour			
1.	LS	Labrale superius	Midpoint of the vermilion border of the upper lip at the deepest retraction of cupid's bow
2. 3.	rCPH/ lCPH	Right/left christa philtri	Points at the intersection of the vermilion border and the elevated margin of the right and left philtrum
4. 5.	rLS/ lLS	Right/left labrale superius	Midpoint between LS and right/left cheilion (rCH/lCH) at the vermilion border (hypothetically quartering the upper lip)
6. 7.	rLI/ lLI	Right/left labrale inferius	Midpoint between LI and rCH/lCH at the vermilion border (hypothetically quartering the lower lip)
8.	LI	Labrale inferius	Caudalmost midpoint of the vermilion border of the lower lip at the intersection with a straight line SN-LS
Along the center of the face (except LS and LI)			
9.	SN	Subnasale	Most dorsal point of the nasolabial curvature, intersection of the columella with the labial white of the upper lip
10.	SSP	Subspinale (A‘-point)	The most dorsal point of the anterior contour of the upper lip, midpoint between the SN and LS
11.	LS-con	Upper lip point	Most convex center of the upper lip on the straight line SN-LS
12.	LI-con	Lower lip point	Most convex center of the lower lip on the straight line SN-LS
13.	SL	Sublabiale	On the straight line LS-LI with distance |SSP-LS| from LI
14.	SM	Submentale	Most dorsal point of the anterior contour of the lower lip (in supramental fold)
Periphery of the red of the lips			
15. 16.	rSALA/ lSALA	Right/left subalare	Point centrally below the right/left nostril
17. 18.	rLS1/ lLS1	Right/left labrale superius 1	On the straight line rLS-lLI/lLS-rLI with distance |SSP-LS| from rLS/lLS
19. 20.	rLI1/ lLI1	Right/left labrale inferius 1	On the straight line lLS-rLI/rLS-lLI with distance |SSP-LS| from rLI/lLI
Auxiliary points for landmark construction (level of occlusal plane)			
	r1/l1	Right/left point 1	Points at the most lateral aspect of the vermilion border of the right and left corner of the participant’s mouth
	r2/l2	Right/left point 2	Projected buccal cusp tip of 14/24
	r3/l3	Right/left point 3	Projected buccal transverse fissure of 16/26
Buccal area: above the occlusal plane (crown level)			
21. 22.	r1S/ l1S	Right/left point 1 superior	Ablation ⊥ upwards to the straight line r1-r3/l1-l3 through the point r1 or l1 with a distance of 0.5 cm
23. 24.	r2S/ l2S	Right/left point 2 superior	Ablation ⊥ upwards to the straight line r1-r3/l1-l3 through the point r2 or l2 with a distance of 0.5 cm
25. 26.	r3S/ l3S	Right/left point 3 superior	Ablation ⊥ upwards to the straight line r1-r3/l1-l3 through the point r3 or l3 with a distance of 0.5 cm
Buccal area: above the occlusal plane (apical tooth region)			
27. 28.	r1S1/ l1S1	Right/left point 1 superior 1	Ablation ⊥ upwards to the straight line r1-r3/l1-l3 through the point r1 or l1 with a distance of 1.5 cm
29. 30.	r2S1/ l2S1	right/left point 2 superior 1	Ablation ⊥ upwards to the straight line r1-r3/l1-l3 through the point r2 or l2 with a distance of 1.5 cm
31. 32.	r3S1/ l3S1	Right/left point 3 superior 1	Ablation ⊥ upwards to the straight line r1-r3/l1-l3 through the point r3 or l3 with a distance of 1.5 cm
Buccal area: below the occlusal plane (crown level)			
33. 34.	r1I/ l1I	Right/left point 1 inferior	Ablation ⊥ downwards to the straight line r1-r3/l1-l3 through the point r1 or l1 with a distance of 0.5 cm
35. 36.	r2I/ l2I	Right/left point 2 inferior	Ablation ⊥ downwards to the straight line r1-r3/l1-l3 through the point r2 or l2 with a distance of 0.5 cm
37. 38.	r3I/ l3I	Right/left point 3 inferior	Ablation ⊥ downwards to the straight line r1-r3/l1-l3 through the point r3 or l3 with a distance of 0.5 cm
Buccal area: below the occlusal plane (apical tooth region)			
39. 40.	r1I1/ l1I1	Right/left point 1 inferior 1	Ablation ⊥ downwards to the straight line r1-r3/l1-l3 through the point r1 or l1 with a distance of 1.5 cm
41. 42.	r2I1/ l2I1	Right/left point 2 inferior 1	Ablation ⊥ downwards to the straight line r1-r3/l1-l3 through the point r2 or l2 with a distance of 1.5 cm
43. 44.	r3I1/ l3I1	Right/left point 3 inferior 1	Ablation ⊥ downwards to the straight line r1-r3/l1-l3 through the point r3 or l3 with a distance of 1.5 cm

Face scans were taken without veneers and with veneers either in the upper jaw, in the lower jaw, or in both jaws. Participants were randomly assigned to one of four groups depending on the sequence of veneer positioning in the maxilla, mandible, and upper and lower jaws (Table 2) to avoid any systematic bias resulting from the sequence of veneer placement.

**Table 2:** Scan sequence

**1. Basic scans for all groups before the experimental scan sequence**			
1 × scan in maximum intercuspation with retracted lips (ICPS)			
3 × rest scan (RS1-3) with RS1 serving as the reference for alignment of all further scans			
**2. Experimental scan sequence**			
Group 1	Group 2	Group 3	Group 3
Thinnest veneer first (1 mm → 4 mm)	Thickest veneer first (4 mm → 1 mm)	Thinnest veneer first (1 mm → 4 mm)	Thickest veneer first (4 mm → 1 mm)
1. UI	1. UI	1. LI	1. LI
2. UI + LI	2. UI + LI	2. UI + LI	2. UI + LI
3. LI	3. LI	3. UI	3. UI

*UI* Veneers on the upper four incisors only, *LI* Veneers on the lower four incisors only, *UI* + *LI* Veneers on both upper and lower incisors

Participants were randomly assigned to their group by drawing labelled envelopes. T.M.P. inserted and scanned all veneers so was aware of the group allocation. However, the participants were not aware of the differences between groups or their group allocation, so corresponding bias was avoided.

Veneers were adhesively bonded to the dry labial crown surfaces using Transbond™ XT (3 M, Saint Paul, USA) without enamel preconditioning. This ensured secure veneer attachment and uncomplicated removal without damaging the enamel surface.

Facial morphology is affected by posture, mimic gestures, motions, and breathing [4, 26], so participants were given specific instructions to standardize these variables before each scan. Since veneers potentially impede lip closure, participants were asked to keep their lips as relaxed as possible, which may have resulted in lip incompetence (at least with the thicker labial veneers inserted). To ensure the mandible was relaxed in a resting position, each participant was asked to count from 1 to 5. Then, 2 ml water was inserted into the mouth using a syringe and the participant was asked to swallow this after five seconds.

**Fig. 3:**
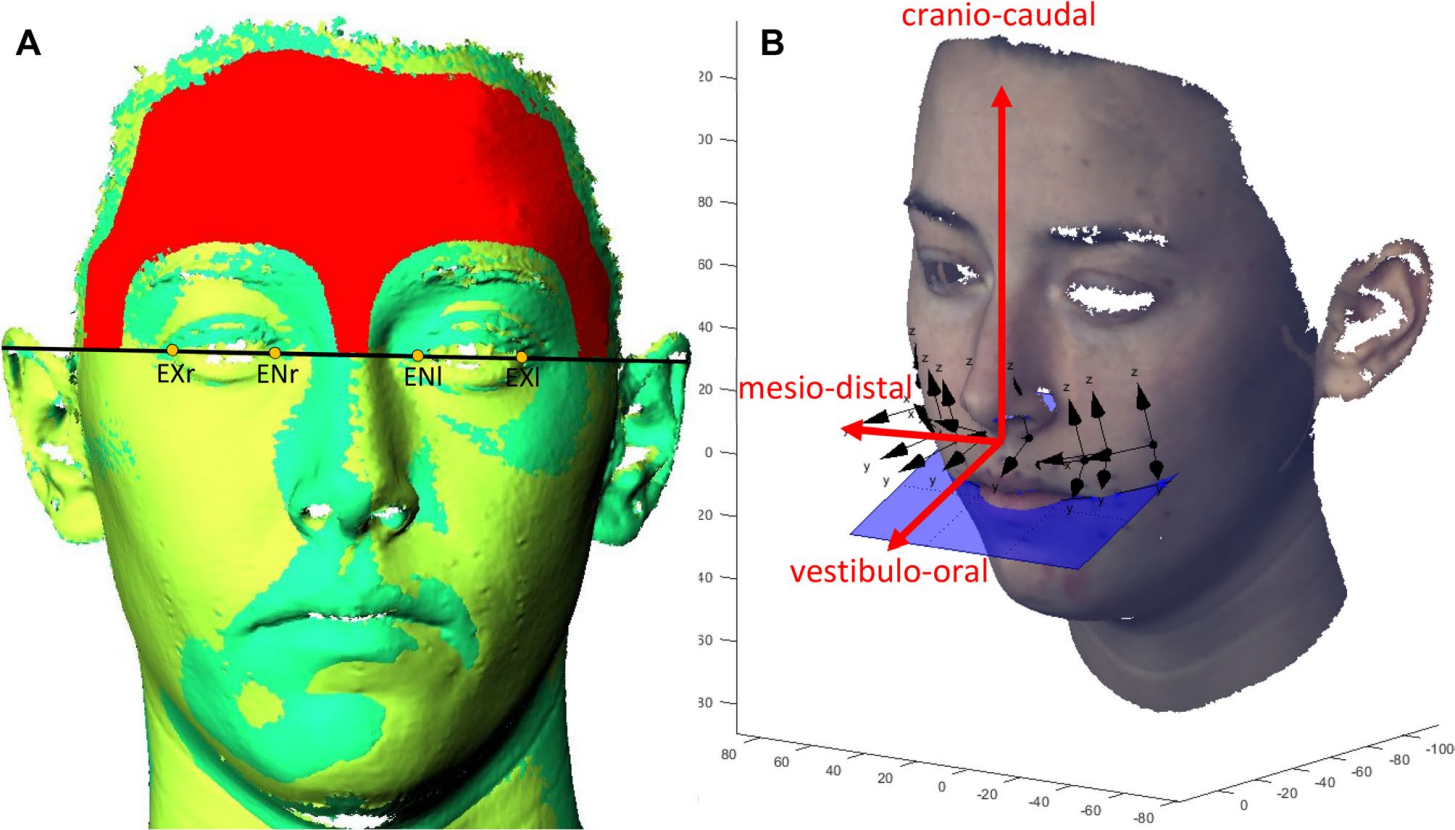
**A** Prealignment was performed by a best-fit method implemented in the scanner software. Fine alignment was performed using predefined surfaces (red area) of the forehead, temple, and nose bridge above a line running through right and left exocanthion and endocanthion. **B** Landmark-specific coordinate system with a curved horizontal axis reflecting the varying vestibulo-oral directions. Black dots = landmarks; arrows = x-/y-/z-axes; blue plane = projected occlusion plane through the landmarks r3, r2, r1, l1, l2, l3

To determine the lip thickness before taking the experimental scans with veneers, one scan was performed in maximum intercuspation with the lips gently retracted (ICPS). After this, we performed three face scans with the lower jaw in its resting position without any occlusal contacts (RS). Participants were instructed to close their lips gently without active contraction, ensuring a relaxed and natural lip posture. The first RS (RS1) served as the reference for aligning all further scans in a two-phase process (Software Optocat) (Fig. 3A).

Except for one ICP scan, each scan was repeated three times resulting in a total of 40 scans per participant (1× ICPS and 3 × RS and 3 × for each of the three veneer combinations in four different thicknesses). This large dataset enabled detection of consistent trends and interindividual variability despite the relatively small sample size. The marked perioral landmarks remained stable during the entire procedure. To quantify the upper lip thickness, the digital maxillary arch model was first integrated into the ICP scan by superposing the labial surfaces of the upper anterior teeth. In a second step, these scans were superimposed with the RS1 scan with relaxed lips. Lip thickness then was measured along the Y-axis at the labrale superius (LS) and left and right christa philtri (lCPH and rCPH) up to the first contact point on the integrated jaw scan.

An independent coordinate system was defined for each landmark (Fig. 3B), with the z-axis in the cranio-caudal direction pointing vertically upward, the y-axis in the vestibulo-oral direction pointing vestibularly, and the x-axis in the mesio-distal direction pointing towards the patient's right. To define the coordinate systems, an averaged plane was fitted through the landmarks at the height levels H2 and H3 (Table 3). 

**Table 3:** Division of buccal landmarks in four levels to create a landmark-specific coordinate system

**1. Level H1**	*r* + l 3S1	*r* + l 2S1	*r* + l 1S1	*r* + l SALA
**2. Level H2**	r + l 3 S	r + l 2 S	r + l 1 S	r + l CPH
**3. Level H3**	r + l 3I	r + l 2I	r + l 1I	r + l LI1
**4. Level H4**	r + l 3I1	r + l 2I1	r + l 1I1	

r+l: right and left; each landmark is described in Table 1

This plane established the common horizontal orientation, with the z-axis positioned perpendicular to it. Additionally, a third-degree polynomial curve was fitted through the landmarks at each height level. The tangential at each landmark determined the corresponding x-direction, while the y-axis was derived in accordance with the conventions for right-handed coordinate systems. The 44 landmarks of each of the 39 scans (not for ICPS) were manually clicked in a fixed order by the investigator using a standard computer mouse (Software Matlab, Mathworks, Nattick, USA). Mean values of the three scan repetitions were calculated for each landmark to account for intraindividual variability in landmark placement.

### Statistical analysis

Variability of the three scan repetitions was determined by calculating the standard deviation for each participant, veneer combination, veneer thickness, landmark, and direction (x, y, and z). These standard deviations were combined across participants using linear mixed-effects models with participants as random effects.

Linear mixed-effects regression analysis was used to predict the extent of soft-tissue change. These models estimated the interrelation between veneer thickness and soft-tissue displacement in a separate model for each landmark and veneer combination. Since the landmark position did not change with a veneer thickness of 0 mm, all linear functions were forced to run through zero, but included a random intercept for participants to adjust for repeated measurements. We also estimated region-specific grouped landmark averages. Models’ assumptions were controlled using residual and quantile-quantile plots.

Mixed-effects model analysis was also used to determine how lip thickness affected soft-tissue changes at the LS, rCPH, and lCPH using lip thickness and veneer thickness as fixed effects. An interaction term was included for both variables, and participants were included as random intercepts.

## Results

**Fig. 4:**
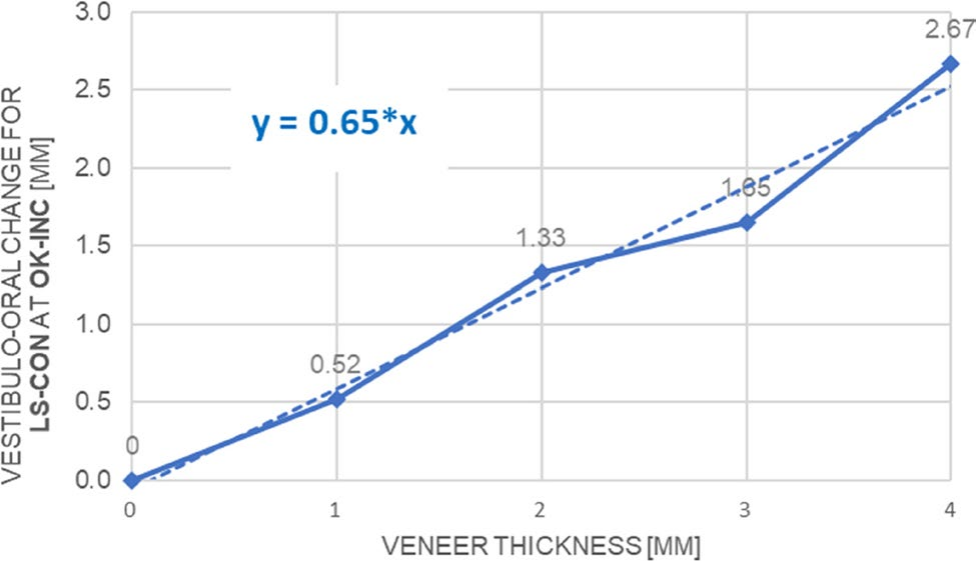
A straight line was fitted to represent the vestibulo-oral soft tissue displacement (in mm) of the LS-con landmark while simulating antepositioning of the upper incisors in relation to veneer thickness (in mm)

All participants completed the study and their data were included in the analyses. The increase in lip and perioral soft-tissue changes was nearly linear with increasing veneer thickness for all landmarks, so we quantitatively characterized soft-tissue changes per millimetre of hard-tissue change for each landmark using linear fitting. For this, the linear functions were forced through zero as the landmarks should be at zero without veneers. Accordingly, the LS-con landmark was displaced 0.65 mm anteriorly per millimetre of veneer thickness (Fig. 4).

Table 4 shows the vestibulo-oral soft-tissue changes at all landmarks resulting from veneers on the upper and/or lower incisors. This interrelationship is shown graphically for the LS-con landmark in Fig. 4.

**Table 4:** Mean vestibulo-oral displacement rates

Landmarks and veneer configurations		Veneer thickness [mm]			
		1	2	3	4
Right point 3 superior 1 (r3S1)	at UI	−0.162	−0.275	−0.196	−0.221
	at LI	−0.123	−0.287	−0.076	−0.175
	at UI + LI	−0.132	−0.432	−0.426	−0.48
Right point 3 superior (r3S)	at UI	−0.169	−0.308	−0.323	−0.221
	at LI	−0.061	−0.325	−0.127	−0.192
	at UI + LI	−0.153	−0.431	−0.419	−0.54
Right point 3 inferior (r3I)	at UI	−0.107	−0.261	−0.312	−0.259
	at LI	−0.072	−0.302	−0.187	−0.292
	at UI + LI	−0.176	−0.328	−0.242	−0.454
Right point 3 inferior 1 (r3I1)	at UI	−0.026	−0.197	−0.28	−0.273
	at LI	−0.015	−0.208	−0.131	−0.211
	at UI + LI	−0.107	−0.217	−0.097	−0.386
Right point 2 superior 1 (r2S1)	at UI	−0.042	−0.09	0.054	0.176
	at LI	0.023	−0.16	0.116	0.135
	at UI + LI	−0.03	−0.209	−0.133	0.021
Right point 3 superior (r2S)	at UI	−0.047	−0.02	0.091	0.358
	at LI	0.031	−0.174	0.066	0.076
	at UI + LI	0.018	−0.133	−0.018	0.019
Right point 2 inferior (r2I)	at UI	−0.055	0.047	0.125	0.302
	at LI	0.054	−0.086	−0.002	−0.12
	at UI + LI	−0.053	0.003	0.252	0.011
Right point 2 inferior 1 (r2I1)	at UI	0.036	0.01	0.07	0.174
	at LI	0.103	0.059	0.065	0
	at UI + LI	−0.045	0.08	0.28	0.046
Right point 1 superior 1 (r1S1)	at UI	0.013	0.066	0.172	0.455
	at LI	0.104	0.005	0.112	0.278
	at UI + LI	0.054	0.009	0.096	0.376
Right point 1 superior (r1S)	at UI	−0.072	0.221	0.445	1.055
	at LI	0.086	0.01	0.112	0.178
	at UI + LI	0.143	0.207	0.577	0.77
Right point 1 inferior (r1I)	at UI	−0.022	0.301	0.502	1.079
	at LI	0.069	0.086	0.043	0.192
	at UI + LI	0.2	0.426	0.921	0.853
Right point 1 inferior 1 (r1I1)	at UI	0.016	0.065	0.147	0.389
	at LI	0.19	0.135	0.11	0.184
	at UI + LI	0.115	0.174	0.502	0.404
Right labrale superius 1 (rLS1)	at UI	0.309	0.77	0.944	1.628
	at LI	0.118	0.07	0.075	0.218
	at UI + LI	0.367	0.7	1.136	1.474
Right labrale inferius 1 (rLI1)	at UI	0.062	0.567	0.655	1.016
	at LI	0.29	0.419	0.524	0.796
	at UI + LI	0.376	0.745	1.379	1.524
Right labrale superius (rLS)	at UI	0.423	1.224	1.533	2.361
	at LI	0.144	0.128	0.163	0.214
	at UI + LI	0.549	1.108	1.76	2.029
Right labrale inferius (rLI)	at UI	0.409	0.99	1.297	1.578
	at LI	0.386	0.697	0.916	1.297
	at UI + LI	0.696	1.283	2.07	2.293
Right subalare (rSALA)	at UI	0.106	0.209	0.207	0.378
	at LI	0.064	−0.015	0.07	0.104
	at UI + LI	0.039	0.071	0.166	0.227
Right christa philtri (rCPH)	at UI	0.385	1.036	1.182	1.912
	at LI	0.078	0.171	0.235	0.295
	at UI + LI	0.343	0.877	1.351	1.568
Subnasale (SN)	at UI	−0.14	0.078	0.076	0.345
	at LI	0.282	0.001	0.057	0.086
	at UI + LI	0.08	0.258	0.296	0.208
Subspinale (SSP)	at UI	0.315	0.657	0.788	1.373
	at LI	0.141	0.234	0.286	0.305
	at UI + LI	0.298	0.618	0.935	1.187
Labrale superius (LS)	at UI	0.521	1.245	1.462	2.31
	at LI	0.147	0.229	0.303	0.334
	at UI + LI	0.466	1.148	1.717	1.802
Labrale superius convex (LS-con)	at UI	0.524	1.328	1.65	2.666
	at LI	0.283	0.19	0.454	0.357
	at UI + LI	0.668	1.349	2.121	2.098
Labrale inferius convex (LI-con)	at UI	0.689	1.453	1.797	2.184
	at LI	0.451	0.755	1.38	1.937
	at UI + LI	0.988	1.709	2.681	3.056
Labrale inferius (LI)	at UI	0.081	0.626	1.021	1.375
	at LI	0.366	0.636	1.213	1.715
	at UI + LI	0.69	1.207	1.931	2.269
Sublabiale (SI)	at UI	−0.084	0.261	0.337	0.719
	at LI	0.45	0.443	0.963	1.453
	at UI + LI	0.595	0.724	1.223	1.37
Submentale (SM)	at UI	0.021	0.413	0.346	0.678
	at LI	0.435	0.221	0.528	0.925
	at UI + LI	0.522	0.512	0.878	0.91
Left subalare (lSALA)	at UI	0.08	0.19	0.213	0.583
	at LI	0.12	0.111	0.091	0.267
	at UI + LI	0.174	0.361	0.454	0.53
Left christa philtri (lCPH)	at UI	0.369	1.029	1.199	1.962
	at LI	0.18	0.222	0.237	0.311
	at UI + LI	0.416	0.98	1.466	1.631
Left labrale superius (lLS)	at UI	0.461	1.236	1.635	2.445
	at LI	0.084	0.142	0.174	0.212
	at UI + LI	0.557	1.192	1.853	2.102
Left labrale inferius (lLI)	at UI	0.492	1.115	1.394	1.812
	at LI	0.293	0.747	1.026	1.526
	at UI + LI	0.707	1.33	2.249	2.569
Left labrale superius 1 (lLS1)	at UI	0.373	0.756	1.107	1.71
	at LI	0.09	0.085	0.081	0.173
	at UI + LI	0.422	0.776	1.202	1.511
Left labrale inferius 1 (lLI1)	at UI	0.149	0.731	0.891	1.275
	at LI	0.193	0.48	0.646	1.102
	at UI + LI	0.396	0.764	1.643	1.926
Left point 1 superior 1 (l1S1)	at UI	0.086	0.259	0.307	0.561
	at LI	0.087	0.029	0.179	0.335
	at UI + LI	0.147	0.235	0.299	0.505
Left point 1 superior (l1S)	at UI	0.12	0.565	0.845	1.336
	at LI	0.123	0.038	0.224	0.372
	at UI + LI	0.323	0.446	0.889	1.113
Left point 1 inferior (l1I)	at UI	0.166	0.766	0.882	1.295
	at LI	0.192	0.118	0.411	0.373
	at UI + LI	0.397	0.508	1.124	1.168
Left point 1 inferior 1 (l1I1)	at UI	0.066	0.314	0.331	0.489
	at LI	0.209	0.114	0.12	0.262
	at UI + LI	0.209	0.148	0.586	0.647
Left point 2 superior 1 (l2S1)	at UI	−0.123	−0.202	−0.237	−0.053
	at LI	−0.119	−0.203	0.061	0.015
	at UI + LI	−0.09	−0.274	−0.261	−0.172
Left point 2 superior (l2S)	at UI	−0.007	0.023	−0.002	0.224
	at LI	0.005	−0.163	0.104	0.124
	at UI + LI	0.057	−0.154	−0.024	0.04
Left point 2 inferior (l2I)	at UI	−0.048	0.163	0.107	0.277
	at LI	−0.019	−0.151	0.027	0.085
	at UI + LI	0.017	−0.093	0.141	0.098
Left point 2 inferior 1 (l2I1)	at UI	0.062	0.235	0.13	0.227
	at LI	0.091	−0.02	0.084	0.082
	at UI + LI	0.078	−0.055	0.325	0.224
Left point 3 superior 1 (l3S1)	at UI	−0.169	−0.351	−0.39	−0.34
	at LI	−0.148	−0.318	−0.13	−0.254
	at UI + LI	−0.154	−0.423	−0.494	−0.606
Left point 3 superior (l3S)	at UI	−0.107	−0.284	−0.309	−0.274
	at LI	−0.089	−0.315	−0.046	−0.184
	at UI + LI	−0.12	−0.394	−0.381	−0.515
Left point 3 inferior (l3I)	at UI	−0.057	−0.134	−0.134	−0.133
	at LI	0.005	−0.209	0.008	−0.141
	at UI + LI	−0.072	−0.321	−0.165	−0.388
Left point 3 inferior 1 (l3I1)	at UI	−0.037	−0.072	−0.055	−0.212
	at LI	0.018	−0.216	−0.058	−0.143
	at UI + LI	−0.124	−0.274	−0.086	−0.302

Displacement rate [mm] for all landmarks for the simulation of protrusion of the upper incisors (UI), lower incisors (LI) and both upper and lower incisors (UI+LI) at different veneer thicknesses (1 to 4 mm)

**Fig. 5:**
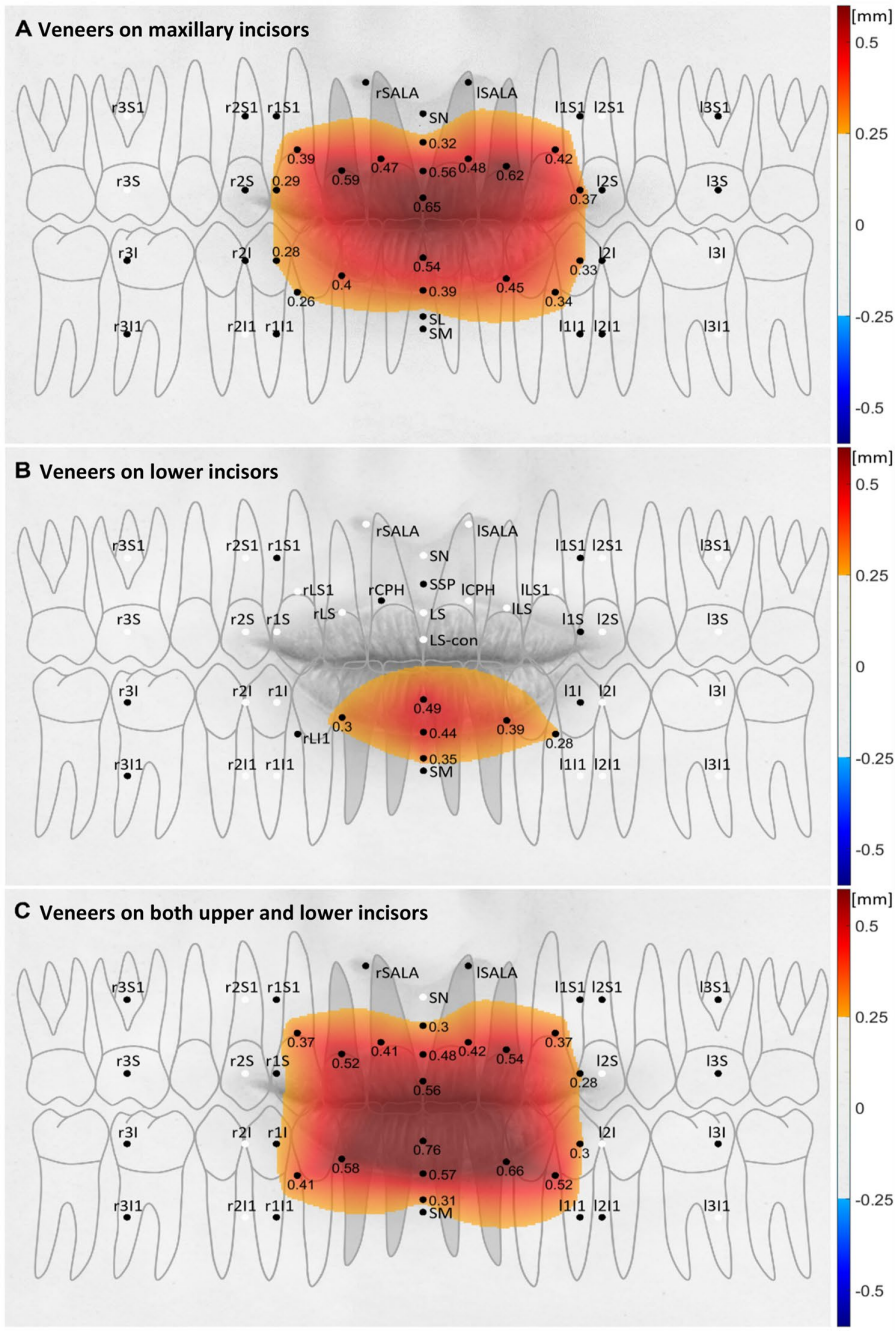
Interpolated colour-coded **vestibulo-oral** soft tissue displacement with veneers on maxillary incisors **A**, mandibular incisors **B**, and both upper and lower incisors **C**. Values are mean values averaged over displacement rates of all veneer thicknesses (1, 2, 3 and 4 mm) across all participants (*n* = 10)

Putting veneers on maxillary incisors only displaced the upper lip area (rLS, LS, LS-con, lLS) and the centre of the lower lip (LI-con) anteriorly (Fig. 5A).

Positioning isolated veneers on mandibular incisors resulted in a clinically relevant anterior displacement in the lower lip area only (rLI, LI-con, LI, lLI, lLI1, SL). LI-con showed an anterior displacement rate of 0.5 mm/mm (CI: 0.38–0.61). This displacement decreased successively in the surrounding regions (Fig. 5B). No significant changes were found in the non-intervention area of the cheeks (r3S1, r2S1, r3S, r2S, r1S, r2I, r2I1, r1I, l1I1, l2I1, l3I1, l3I, l2S, l3S, l2S1, l3S1) or between the nose and upper lip (rLS1, rLS, rSALA, SN, LS, LS-con, lSALA, lCPH). When veneers were placed on mandibular incisors only, the mean displacement rate was 0.41 mm/mm (CI: 0.36–0.47) for the whole lower lip and just 0.05 mm/mm (CI: 0.02–0.09) for the whole upper lip (Table 5 A).

Positioning veneers on both the upper and lower incisors had distinct effects on both lips. Displacement of the lower lip at LI-con increased to 0.76 mm/mm (CI: 0.65–0.87) when additional veneers were positioned on the maxillary incisors. In contrast, anterior displacement of the upper lip at LS-con decreased to 0.56 mm/mm (CI: 0.47–0.65) when additional veneers were positioned on the mandibular incisors. As shown in Fig. 5C, when veneers were positioned on both maxillary and mandibular incisors, soft-tissue changes of ≥ 0.5 mm were limited to the area of the lower lip (rLI, LI-con, LI, lLI, LI1l) and the inferior part of the upper lip (rLS, LS-con, lLS). Under these conditions, the mean displacement rate was 0.63 mm/mm (CI: 0.57–0.69) for the whole lower lip and 0.48 mm/mm (CI: 0.44–0.52) for the upper lip (Table 5 A).

The greatest vertical changes were observed following veneer placement on upper incisors only. Rates of cranial displacement were 0.36 mm/mm (CI: 0.28–0.43) for lCPH, 0.32 mm/mm (CI: 0.25–0.40) for rCPH, 0.31 mm/mm (CI: 0.24–0.39) for LS, and 0.23 mm/mm (CI: 0.16–0.30) for LS-con. Vertical displacement was restricted to the medial area in the lower lip and decreased considerably in lateral areas. Caudal displacement was 0.37 mm/mm (CI: 0.26–0.48) for LI-con and 0.32 mm/mm (CI: 0.22–0.42) for LI. Positioning veneers on only the maxillary incisors resulted in a mean upward positioning of the whole upper lip of 0.33 mm/mm (CI: 0.29–0.36), and a mean downward positioning of the whole lower lip of 0.23 mm/mm (CI: 0.17–0.29) (Table 5B).

Simulated protrusion with lower incisor veneers caused vertical displacements in the midsagittal plane of the lower lip. Caudal displacement was 0.34 mm/mm (CI: 0.24–0.44) for SL, followed by 0.28 mm/mm (CI: 0.16–0.41) for LI-con, 0.23 mm/mm (CI: 0.10–0.36) for LI, and 0.17 mm/mm (CI: 0.07–0.28) for SM. No significant changes were observed in the upper lip (Table 5B).

As for vestibulo-oral changes, veneers on both the upper and lower incisors resulted in contrasting vertical displacement of both lips. In the lower lip, displacement as additional veneers were positioned on the maxillary incisors increased further to 0.66 mm/mm (CI: 0.52–0.79) for LI-con, 0.67 mm/mm (CI: 0.54–0.80) for LI, 0.53 mm/mm (CI: 0.39–0.66) for SL, and 0.27 mm/mm (CI: 0.13–0.41) for SM. In the upper lip, vertical displacement as additional veneers were positioned in the opposite jaw decreased to 0.21 mm/mm (CI: 0.14–0.29) for rCPH, 0.18 mm/mm (CI: 0.10–0.25) for lCPH, 0.15 mm/mm (CI: 0.06–0.24) for LS, and 0.12 (CI: 0.06–0.20) for LS-con.

In the mesiodistal direction, clinically relevant changes were only found in r1I and l1I, which were positioned at the crown level of the first right and left lower premolar, and in two veneer configurations. Positioning veneers on maxillary incisors resulted in a distal displacement rate of 0.26 mm/mm (CI: 0.15–0.37) for r1I and 0.29 mm/mm (CI: 0.20–0.39) for l1I. When veneers were positioned on both the upper and lower incisors, the distal displacement rates increased to 0.34 mm/mm (CI: 0.25–0.44) for r1I and 0.37 mm/mm (CI: 0.27–0.46) for l1I. No relevant changes were observed for the whole upper and lower lip (Table 5C).

**Table 5:**
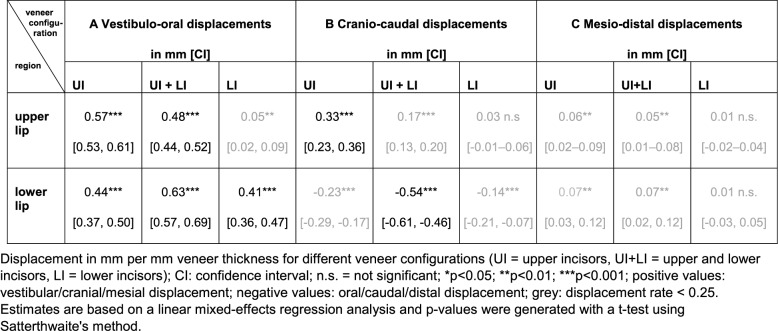
Mean displacement of the whole upper/lower lip

Linear mixed-effects models showed that participants with thicker lips had significantly reduced forward positioning of the lips per millimetre of veneer thickness *(P <* 0.05). This effect was expressed numerically by the interaction factor, which describes the difference in soft-tissue displacement (in mm) per millimetre difference in lip and veneer thickness. For instance, combined simulated protrusion of upper and lower incisors gave a factor of interaction of − 0.08 mm at LS (CI: −0.13 to − 0.02 mm, *P* < 0.01). Hence, increasing lip thickness at this landmark by 1 mm significantly attenuates vestibulo-oral soft-tissue deflection by 8%. The interaction factor was − 0.04 at rCPH (CI: −0.07 to − 0.01 mm, *P* < 0.05) and − 0.04 at lCPH (CI: −0.08 to − 0.01 mm, *P* < 0.05).

Lip thickness ranged from 9.9 mm to 15.0 mm at LS in our participants. Considering the veneer thicknesses ranged from 1 mm to 4 mm, the estimated vestibulo-oral lip displacement at LS was 1.92 mm for the participant with the thinnest lips and 0.74 mm for the participant with the thickest lips. Corresponding values for landmarks rCPH and lCPH were respectively 1.62 mm and 1.54 mm for the participant with the thinnest lips and 0.65 mm and 0.79 mm for the participant with the thickest lips.

Lip thickness also significantly affected vertical soft-tissue displacements at LS. Isolated veneer positioning on the upper incisors gave an interaction factor of − 0.05 (CI: −0.1 to − 0.01, *P* < 0.05). Hence, increasing lip thickness at this landmark by 1 mm significantly attenuated vertical soft-tissue deflection by 5%.

## Discussion

Several studies have investigated the effects of simulated maxillary incisor protrusion and anterior tooth retraction following premolar extraction on perioral soft tissues. This experimental study on human participants presents a novel approach to investigating the effects of isolated and combined upper and lower incisor protrusion on soft tissues in the frontal and buccal area.

We simulated changes in incisor positions using digitally designed 3D-printed acrylic veneers with incremental thicknesses of 1–4 mm to minimize manufacturing-related error [9]. Changes in perioral soft tissues were evaluated by taking 3D face scans both with and without veneers. No subjective aesthetic evaluation was performed. All soft-tissue changes were assessed based on objective 3D measurements using 44 predefined anatomical landmarks. These landmarks were marked directly on the skin and monitored throughout, in contrast to other studies that marked landmarks retrospectively in the scans. Our 3D analysis of soft-tissue displacements used a novel landmark-specific coordinate system whereas previous studies have used a conventional coordinate system. The differences between these approaches affect the evaluation of the horizontal soft-tissue displacements; therefore, corresponding values should be compared with those from other studies with caution.

Perioral soft-tissue changes were highest in the vestibulo-oral dimension, followed by the cranio-caudal and mesio-distal dimensions, in agreement with findings from another study [6]. This is likely because we simulated the vestibular displacement of the incisor crowns. Soft-tissue changes of more than one millimetre are currently classified as clinically relevant in line with previous literature suggesting that soft-tissue changes of this magnitude are typically perceptible in the perioral region [27–29].

Our results show that soft-tissue changes after incisor movement extend beyond the vermilion borders. This justifies the use of a dense landmark approach: by analysing 44 defined landmarks, we were able to capture region-specific patterns—such as pronounced vestibulo-oral displacement in the median upper lip that diminished laterally—which might have been missed with fewer landmarks. To our knowledge, few comparable studies have analysed soft-tissue changes using such a high number of defined landmarks, enabling us to perform a more precise and clinically relevant analysis of soft-tissue responses.

This analysis revealed no clinically relevant soft-tissue changes lateral to the oral commissures with any veneer combination, possibly because these areas were further away from the added veneers. Another explanation could be the histological differences mesial and distal to the nasolabial fold [30–33].

In agreement with our findings, previous studies simulating incisor protrusion found greatest displacement at the vermilion border with a handmade wax [6], a handmade acrylic resin veneer [9], and films [24] of different thicknesses. Kim et al. found that 1-mm thick films induced similar soft-tissue changes as observed in our study (0.54 mm for LS) [24], and Au et al. found that 2-mm thick veneers induced similar advancement per millimetre of simulated tooth movement as we did (0.66 mm for LS, 0.51 mm for LI, 0.54 mm for rCPH, and 0.55 mm for lCPH) [6, 8]. In the study by Kim et al., the extent of perioral soft tissue change above the vermilion border was slightly less than 50% of simulated incisor advancement [24], suggesting that the extent of vestibulo-oral changes is dependent on the vertical position of the landmark, with changes decreasing towards the nose [24]. In addition, Rosati et al. found no significant soft-tissue changes for landmarks further from the lip with their acrylic resin veneers [9].

Positioning veneers on mandibular incisors only caused anterior displacement in the lower lip area. These changes were greatest in the centre of the lower lip and were not clinically relevant in the upper lip. The lower lip has been reported to be more sensitive to movement of the lower incisors than the upper lip is [2, 14]. However, we cannot directly compare our findings with these studies because they did not limit treatment to the lower jaw in their included patients.

In our study, displacement of the lower lip increased when veneers were positioned on both upper and lower incisors, whereas anterior displacement of the upper lip decreased when veneers were placed on both upper and lower incisors. No previous studies have simulated upper and lower incisor protrusion simultaneously. Studies investigating anterior tooth retraction after the extraction of four premolars reported similar results but in the posterior direction. One millimetre of combined upper and lower incisor retraction resulted in a posterior movement of the upper lip of 0.41 to 0.53 mm and of the lower lip of 0.22 to 0.82 mm [19, 20]. This wide range is likely due to methodological heterogeneity across studies, including differences in imaging, alignment and evaluation techniques. Shen et al. reported that changes in the lower lip were four times greater than those in the upper lip [23]. In agreement with our findings, another study showed that lip movement was greater within the lips than at the lip border [19], and backward movement of the lower lip was almost double in the greatest bulge area than that seen along the lip border [19].

In general, lower lips react more sensitively [14, 19, 22] and more predictably [8] to incisor movement than upper lips do, although the interindividual variability is high [7, 8]. This could be because upper lip movement is a multifactorial process [7, 14, 19], whereas lower lip movement is mainly influenced by vestibulo-oral movement of mandibular incisors [7, 14, 19] and lip thickness before treatment [7].

Simulated upper incisor protrusion resulted in cranial displacement of the upper lip and caudal displacement of the lower lip. These results are in concordance with the findings of Au et al. and Kim et al. [6, 24], who reported similar rates of approximately 30% of simulated upper incisor protrusion for both LS and LI. To the best of our knowledge, our study is the first to investigate vertical lip displacement following isolated mandibular incisor protrusion and combined maxillary and mandibular incisor protrusion. We observed that placing veneers on only lower incisors had no vertical effect on the upper lip, whereas placing veneers on both the upper and lower incisors had a distinct vertical effect on both lips. Similar to the vestibulo-oral effects we observed, vertical displacement of the lower lip increased whereas vertical displacement of the upper lip decreased. The vertical displacement directions and rates that we observed for the upper and lower lips are in concordance with those reported in premolar extraction studies [19, 21]. However, there are discrepancies between studies. Ahn et al. observed comparable values for upper (≈ 0.1 mm/mm) and lower (≈ 0.6 mm/mm) lip movements [19], whereas Baek et al. reported larger movement of the upper lip (≈ 0.3 mm/mm) than the lower lip (≈ 0.2 mm/mm) [21]. In contrast, Shen et al. found no vertical changes for their landmarks [23]. This might be because Shen et al. aligned their scans using a best-fit algorithm over the entire surface, which could have masked vertical soft-tissue changes.

Half of the participants in our study had an incompetent resting lip posture after the veneers were placed, so we may have overestimated the vertical displacement of the landmarks. This is particularly important when comparing lip retraction after premolar extractions, where opposing soft-tissue movements take place. Most of the participants with lip incompetence were female and the incompetence was already observed when the 2-mm thick veneers were placed. In contrast, lip incompetence in male participants was only observed with 4-mm thick veneers and never with the pure LI configuration. These observations suggest that perioral soft tissues are less resilient to lip incompetence in women than in men, leading to increased lip protrusion. In the mesiodistal direction, clinically relevant changes were only observed in the corners of the mouth with veneers either on only upper incisors or on both upper and lower incisors. These findings agree with those of Ahn et al., who also found most transversal changes in the lateral corners of the mouth [19]. In support of this, Au et al. also showed transverse displacements of < 1.0 mm at the vermilion border even for 6-mm advancement [6]. In concordance with the findings of Au et al. and Kim et al. [6, 24], we observed that more lateral landmarks at the vermilion border had greater lateral displacement.

We observed a nearly linear interrelation between changes in hard and soft tissues, unlike previous reports that soft-tissue responses to simulated protrusion are either nonlinear [6, 24] or linear only within a limited range of movement [7] with the relative response decreasing as the film [24] or wax [6] thickness increased. This discrepancy could be due to inaccuracies in the manufacturing of the veneers. This error may have been minimized in our study by the novel approach of digitally modelling and 3D printing the veneers [9].

Our findings on the association between upper incisor movement and lip movement were consistent with those of other studies. In concordance with our findings, the extent of upper lip movement was approximately linear to the upper incisor retraction after premolar extractions [7, 14, 22]. Furthermore, the relationship we observed between upper incisor advancement and lower lip movement was also consistent with that observed in other studies [6, 8, 9]. In premolar extraction studies, soft-tissue movements occurred in the opposite direction to those observed in our simulated protrusion study, so we can only assume that the extent of the soft-tissue changes is the same in both directions. However, the results reported in these studies are very similar to ours, suggesting that comparisons can be drawn in the examined displacement range. In clinical practice, particularly in borderline extraction/non-extraction cases, anticipating the aesthetic consequences of incisor positioning is crucial. Our data suggest that movement of incisors leads to measurable and direction-specific changes in the perioral soft tissues. This detailed quantitative information may help orthodontists to forecast soft-tissue outcomes more accurately and make treatment choices that meet both functional and aesthetic goals.

The extent of soft-tissue changes can be affected by many factors, including age, BMI, ethnicity, gender, and the type of malocclusion [34]. Our results showed that individuals with thicker lips at baseline exhibited less vestibulo-oral and cranio-caudal soft-tissue displacement per millimetre of incisor protrusion. Rather than a confounder, we see this variation as a clinically relevant factor. Using linear mixed-effects models, we quantified this influence: each 1-mm increase in upper lip thickness at baseline reduced soft-tissue displacement by about 8%. This finding shows the importance of considering individual anatomical characteristics in treatment planning and supports the need for an individualized approach when predicting soft-tissue changes.

In this study, the largest lip thickness difference (5.1 mm) and the largest veneer thickness (4 mm) would reduce soft-tissue displacement at LS by 1.63 mm of the expected total 4 mm displacement, which would be of great clinical significance. If we take possible overestimation of the model into account by using the lowest plausible estimate of the model (2.4% to 13.1%), this estimation would still reduce soft-tissue displacement by 0.49 mm. This observation is supported by other studies [7, 35, 36] and might be explained by a “cushioning effect” of the lip soft tissue as studies have shown more pronounced lip changes in individuals with higher lip muscle tonicity [12, 37, 38] and a higher stiffness in thinner lips than in thicker lips [39].

Differences in baseline lip morphology and tonus may also be influenced by age, sex, or individual muscle activity patterns, which could modulate soft-tissue responses [39, 40]. For example, studies have reported that men typically have thicker lips than women [41], and that sex differences in soft tissue thickness exist across the lower face in adults [42]. These variations likely contribute to interindividual differences in displacement patterns and should be considered in personalised treatment planning. One study found that the effect of lip thickness on soft-tissue changes is also affected by the direction of vestibulo-oral movement, with greater effects in patients undergoing protrusion [2]. They showed that a 1-mm greater lip thickness before treatment reduced soft-tissue protrusion by 0.39 mm but slightly increased retrusive soft-tissue changes [2].

Our study has some limitations. One limitation is that the sample size was small with a limited age range and ethnicity (all participants were Central European). Because there were no data on simulated lower or combined upper and lower incisor protrusion, we could not perform sample size calculation prior to the study. Instead, we chose an exploratory design including 10 participants; this size was considered to be feasible based on the high number of measurements we planned to collect from each participant. A further limitation is that lip incompetence may have led to overestimation of vertical soft-tissue changes; however, we conducted an additional model with lip incompetence as a covariate and found less than 5% of the variance (R² < 0.05), suggesting that lip incompetence only has a minor influence on soft-tissue changes. Regarding the type of movement, the printed veneers simulated only a translatory advancement of the incisor crowns, and ignored movement of the roots and alveolar process. Moreover, bonded veneers can only simulate protrusion of teeth, not retrusion. A further limitation is that we only assessed the immediate perioral soft-tissue changes, unlike the gradual orthodontic tooth movement seen in clinical practice, where soft-tissue adaptation and remodeling occur over time. So we cannot draw conclusions about long-term changes. Despite this, our results offer useful baseline data on soft-tissue displacement to support treatment planning. Finally, various methodological errors need to be taken into account, including scan errors, motion artifacts, alignment errors, and inaccuracies in landmark marking. We reduced the error generated while digitizing the landmarks by marking the landmarks once and using the same mark for all scans [9].

## Conclusions

The following conclusions can be drawn from this study:

(i) Simulated isolated protrusion of the four maxillary incisors causes anterior displacement of the upper lip (reflected by LS-con) of 0.65 mm per millimetre of veneer thickness. The displacement rate of LS-con slightly decreased to 0.56 mm/mm when veneers were additionally bonded to the four mandibular incisors.
(ii) Lower lip displacement was significantly larger after combined protrusion of upper and lower incisors (+ 0.76 mm) than after protrusion of lower incisors only (+ 0.50 mm).
(iii) Vertical lip displacement after upper and lower incisor protrusion was 0.12 (0.05–0.20) mm/mm upward positioning of LS-con and 0.66 (0.52–0.79) mm/mm downward positioning of LI-con.
(iv) An 1-mm increase of initial lip thickness at LS reduced soft-tissue displacement by 8% of the simulated tooth movement.
(v) These findings may help to accurately predict and simulate perioral soft-tissue changes following differentiated protrusion of upper and/or lower incisors during orthodontic treatment and orthognathic surgery.

## Data Availability

Data is provided within the manuscript files.
